# Influence of Old Concrete Age, Interface Roughness and Freeze-Thawing Attack on New-to-Old Concrete Structure

**DOI:** 10.3390/ma14051057

**Published:** 2021-02-24

**Authors:** Jingchong Fan, Lili Wu, Bo Zhang

**Affiliations:** 1 School of Mechanics & Civil Engineering, China University of Mining and Technology, Beijing 100083, China; fjcdcumtb@163.com; 2 State Key Laboratory of Hydroscience and Engineering, Tsinghua University, Beijing 100084, China

**Keywords:** new-to-old concrete, interface roughness, splitting tensile strength, freeze-thaw, fractal dimension

## Abstract

The bonding surface structure generated by the repair of concrete structures has been paid more attention as a weak point. The effects of old concrete age, interface roughness, and freeze-thawing (F-T) attack on adhesive interface are comprehensively investigated. In this study, six kinds of interface roughness and five different old concrete age are designed. The interfacial bonding property is mainly evaluated by splitting tensile strength (*f*_ts_). Fractal analysis was used to characterize the interface roughness using laser scanning data. In general, the *f*_ts_ increased with the increasing value of interface fractal dimension. The relationship between *f*_ts_ and fractal dimension value was further analyzed, considering the old concrete ages and the F-T cycles. The results show that the effect of roughness on the bonding property of new-to-old concrete is more significant than the age of old concrete, and the influence of the F-T cycles on the bonding surface is mainly reflected in the initial stage of the F-T deterioration process. The relative dynamic elasticity modulus decreased obviously under F-T cycles, especially for the specimens with low interface roughness. In combination with the results of two non-destructive methods (ultrasonic non-destructive test and relative dynamic elastic modulus test), the larger roughness and the smaller age of old concrete can improve the bond performance.

## 1. Introduction

Identification, evaluation, reinforcement and repair of aging concrete structures has become a popular industry [1]. With the increase of the application scope and field of concrete materials and the improvement of engineering quality requirements, the durability problems of concrete-to-concrete structures are gradually exposed [2,3]. The construction industry in many countries has entered the period of maintenance and renovation [4]. The existing concrete structure is in urgent need of effectively repair for strengthening [5,6]. A bonding interface is thus formed between the repair material and the concrete substrate. The bonding interface is the weakest part in the repaired structure, and the bonding property is critical for the performance of repaired structure [3,4,7,8,9]. Efficient and durable concrete repair can extend the service life of concrete structure and ensure the long-term durability [10,11,12].

The interfacial bonding properties can be obtained by various tests, and the splitting test is the most widely used [12,13,14]. Momayez et al. [8] defined the bond failure as a failure that occurred along the interface and found that the splitting prism specimen failed in bond compared with pull-off, bi-surface shear and slant shear specimen. Therefore, the splitting tensile strength is the most effective characterization of interfacial bonding property. The splitting tensile strength is usually related to interface roughness, age of concrete substrate and other factors [13,15,16]. Karima Gadri et al. [13] carried out the splitting tensile test according to Standard ASTM C496 in which the force applied was parallel to the bond line interface of the composite specimen, and found that the bonding of repair material was more significant in the case of a high roughness substrate. Espeche et al. [14] proved the bonding properties were closely related to the adhesion of repair material and the roughness of the concrete substrate. Guo et al. [17] found that as the surface roughness increased, the proportion of the aggregates of the repair concrete embedded into the concrete substrate interior increased, which resulting in the increase of the effective bonding area, and thus the bonding property was improved.

The concrete structure is subject to the continuous freezing-thawing cycles in the engineering scale, especially in the cold region. The consideration of durability, such as frost resistance, is essential to evaluate the reliability of repaired structures. Guo et al. [17] found that the splitting tensile strength of low and high roughness specimens decreased to 44.2% and 74.5% after 100 times of F-T cycles, respectively. Qian et al. [18] studied the factors affecting the interfacial tensile bond of concrete repair system and found that the splitting tensile strength of adhesive decreased significantly under the freeze-thawing (F-T) cycles. The damage evolution and plasticity development of concrete materials subjected to F-T during the load process are essential to improve the frost durability of concrete structures.

The damage evolution and plasticity development of concrete are the key to improve the durability of concrete structure under F-T cycles [19,20]. Yi et al. [21] reported that that freeze-thawing damage of new-to-old concrete has two stage changes: first stage, F-T damage increases rapidly, mainly caused by the damage of interface; second stage, freeze-thawing damage increases slower, mainly reflects damage of substrate and overlay. Therefore, most of the researches were focused on the constitutive model of concrete materials subjected to freeze-thaw. Sun et al. [22] established the F-T damage constitutive model and proposed a cohesion reduction parameter, which improved the accuracy of damage constitutive models for concrete. Shang et al. [23] investigated the mechanical properties of concrete subjected to F-T under biaxial compression, and a biaxial constitutive model was developed considering the effect of F-T cycle number and the stress ratio. Wang et al. [24] proposed a macro-mesoscopic coupling damage constitutive model, which can evaluate the durability of concrete materials considering the coupling of F-T cycle and loading. Recently, many innovative numerical calculation methods accompanied with new test methods have been utilized to investigate the constitutive model with F-T attack. Berto et al. [25] developed a more comprehensive representation of the mixed-tension domain, and a chemical-mechanical damage model was further established considering both mechanical and environmental aspects. Li et al. [26] defined a mesoscopic damage parameter by following a unique calculation procedure, and the mesoscopic damage constitutive model was proposed based on mercury intrusion porosimetry, differential scanning calorimetry, and F-T tests. Moreover, a series of experiments were carried out to study the constitutive models of different types of concrete materials under F-T action [27,28,29,30].

The above studies showed that the influence of the bond surface roughness on the splitting tensile strength, but only the qualitative variation is analyzed, no quantitative relationship is obtained. F-T deterioration, as major erosion during service, has not been sufficiently considered in the effectiveness of concrete repair structures. It is urgently needed to establish a prediction model for the splitting tensile strength of bonding interface considering the roughness and freezing-thawing action.

In this paper, different interface roughness of the new-to-old concrete was characterized with the fractal dimension *D*. The splitting tensile tests of the specimens with different roughness, different ages of old concrete and different numbers of F-T cycle were carried out to obtain the influence of the factors on the interface bonding property. The relationship between splitting tensile strength and fractal dimension value was further analyzed, considering the old concrete ages and the F-T cycles. In combination with two non-destructive methods (ultrasonic non-destructive test and relative dynamic elastic modulus test), the relationship between splitting tensile strength of new-to-old concrete and the durability was further researched.

## 2. Materials and Methods 

### 2.1. Specimens Preparation

To cast the concrete specimens with same roughness and reduce the discreteness of the test results, a number of plaster models with the same roughness were prepared as follows: the selected fracture surface of concrete specimen was placed in a mold of 100 mm × 100 mm × 100 mm, then the isolation agent was applied evenly on the rough surface, and the plaster was poured on it; after the plaster generated strength, the concrete and plaster model could be separated. The plaster models with the same roughness were made repeatedly. It is necessary to point that the initial concrete specimens with different natural roughness were obtained by splitting tensile test. The standard cubes were used in the experiments with the dimensions of 100 mm × 100 mm × 100 mm. The cubic compressive strength of the old concrete and the new concrete were 30 N/mm^2^ and 35 N/mm^2^, respectively. The type of ordinary Portland cement used was P.O42.5, with the density of 3.16 g/cm^3^. Coarse aggregate is 5–20 mm gravel, and fine aggregate is river sand (fineness modulus = 2.7). The specimens with different ages of the old concrete (3, 14, 28, 60 and 300 days) were also prepared with 95% relative humidity curing environment and a temperature of 20 °C. After casting the new concrete on the old concrete, the new-to-old concrete was cured at 95% relative humidity curing environment and a temperature of 20 °C. The concrete composition of the old concrete and new concrete are shown in Table 1. 

### 2.2. Characterization Method of Interface Roughness 

**Figure 1:**
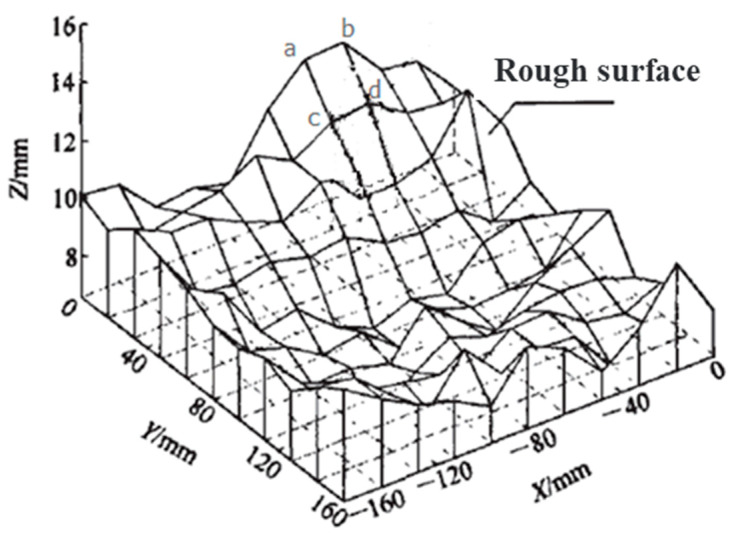
Rough surface diagram of concrete [33].

As the concrete bond surface was fractal like the coastline, fractal dimension was used to characterize the roughness of the interface. In this study, MATLAB (MathWorks, Natick, MA, USA) was used to process the laser scanning data and calculated the fractal dimension values (D) of five different fracture surfaces [31,32]. For laser scanning testing, the three-dimensional coordinate information of the boundary point of the specimen is obtained through the height change between two adjacent scanning points. The least square method was used to fit the analytical formula of the specimen boundary in the laser measuring surface by combining the extreme points of axis coordinates. Furthermore, the position of the coordinate system attached to the workpiece was obtained (relative to the laser measuring coordinate system). Finally, the surface characteristic information is obtained by using the position vector information of the workpiece in the laser measuring coordinate system. The workpiece surface detection error is 0.11 mm, which meets the requirements of specimen surface feature detection. The area of the rough surface should be first calculated. Small squares with side length *δ* cover the whole rough surface, and the height of four corner points of each small square can be obtained through a laser scanner (Figure 1). Then the area of the overall rough surface and the number of squares are obtained [32]. According to fractal theory, side length *δ* and the amount of squares *N*(*δ*) satisfy the following relation:

(1) $$N(\delta )∼\delta^{-D}$$

Finally, the fractal dimension is used to directly represent the roughness of interface. The fractal dimension values (D) of group T, C, Q, S, G, and B are 2.103, 2.237, 2.255, 2.304, 2.315 and 2.341, respectively. 

### 2.3. Splitting Tensile Test

Dynamic and Static Fatigue Testing Machine FLPL204 (Fuller Instrument Technology Co., Ltd., Shanghai, China) was used to obtain the splitting tensile strength. According to GB/T 50081-2002 [34], the splitting tensile test adopted load control, and the loading speed was 0.03 MPa per second. To improve the accuracy of the experiment, each test was carried out three times to calculate the average value. The splitting tensile strength was calculated:

(2) $$f_{ts}=0.85\times \frac{2P}{\pi A}=0.54\times \frac{P}{A}$$

where *f_ts_* is the splitting tensile strength (MPa); 0.85 is the scale conversion coefficient when a non-standard specimen (100 mm × 100 mm × 100 mm) is used; *P* is the failure load of the specimen (N); and *A* is the split surface area (mm^2^).

### 2.4. Ultrasonic Non-Destructive Test

The main working principle of ultrasonic nondestructive testing is to emit ultrasonic waves of different frequencies inside the material or structure. The signal is received and analyzed by the instrument to further understand the damage and defects inside the structure. Due to the complexity of the internal medium of concrete, the transmission of ultrasonic wave in concrete will have different attenuation degrees, which are mainly divided into two forms: absorption and scattering. A large number of experimental studies show that the acoustic attenuation in concrete is mainly scattering attenuation. The relative defect degree *D*_R_ was proposed to characterize the defects inside new-to-old concrete structure, which was expressed as:

(3) $$D_{R}=1-{\left(V_{T}/V_{3}\right)}^{2}$$

where *D_R_* is the relative defect degree, *V*_T_ is the wave velocity of old concrete at *T* days for new-to-old concrete structure (km/s), which satisfied *T* > 3, *V*_3_ was the wave velocity of old concrete at 3 days for new-to-old concrete structure (km/s).

### 2.5. Rapid F-T Cycles Test

According to GB/T 50082-2009 [35], the rapid F-T cycles test was carried out using KDS-28 concrete rapid F-T testing machine. The central temperature of the specimen was −18 ± 2 °C −5 ± 2 °C, and the specimen were always saturated during the F-T process. The rapid F-T tests were conducted on the six groups of specimens, and the ages of the old concrete were 28 and 60 days. The splitting tensile strength of the specimen was measured when the F-T cycles reaches to 0, 5, 10, 20, 30, 45, 60, 80, and 100, respectively.

With the increase of the number of freeze-thaw cycles, the dynamic modulus of each specimen decreases accordingly. The relative dynamic modulus calculation formula is shown as follows:

(4) $$P_{n}=\frac{f_{n}^{2}}{f_{0}^{2}}\times 100\%$$

where *P_n_* is the relative dynamic modulus after n times of F-T cycles, *f_n_* is the transverse fundamental frequency of the specimen after n times of F-T cycles and is the transverse fundamental frequency of the specimen without F-T attack. 

## 3. Results and Discussion

### 3.1. Laser Scanning Results of Bonding Surface 

**Figure 2:**
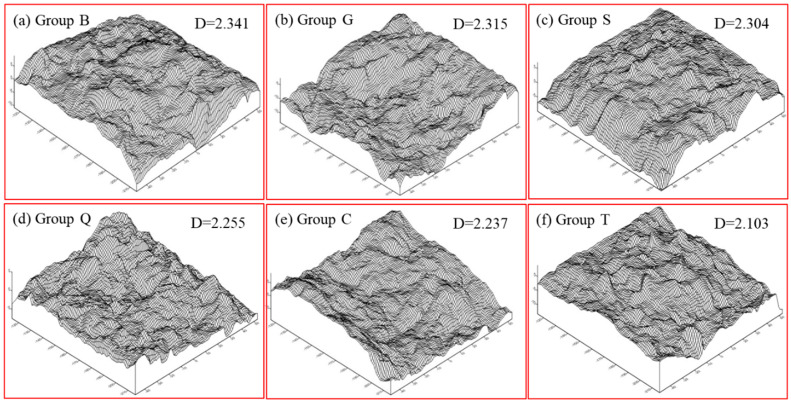
The surface of the new-to-old concrete with different roughness: (**a**) Group B, (**b**) Group G, (**c**) Group S, (**d**) Group Q, (**e**) Group C, (**f**) Group T.

The fracture failure surface of new-to-old concrete occurred along the bond interface basically, and the failure surfaces are shown in Figure 2. For the interface rough specimen in groups B and G, the fracture surface is accompanied with the fracture of cement slurry, the fracture and falling off of aggregate; for the interface smoother specimen in group D, the failure surface is basically intact, but its edges or corners are prone to the phenomenon of aggregate falling off and slurry breaking.

### 3.2. Influence of the Old Concrete Age

**Figure 3:**
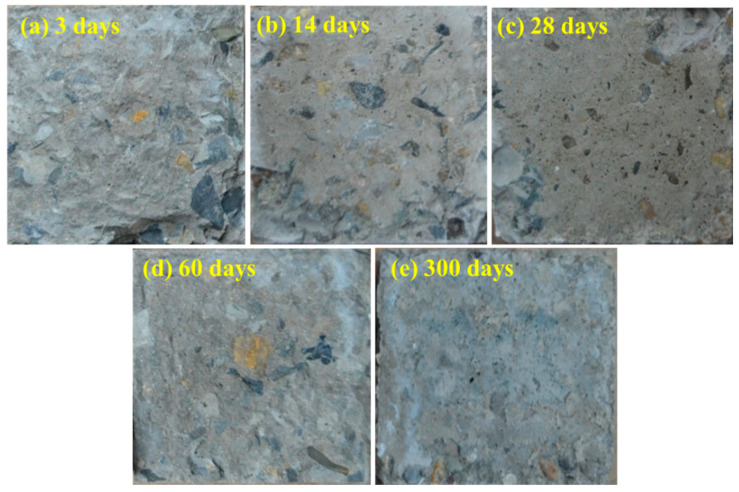
The bonding surface for specimens in group B after splitting tensile tests: (**a**) at 3 days, (**b**) at 14 days, (**c**) at 28 days, (**d**) at 60 days, (**e**) at 300 days.

The splitting tensile tests were carried out on the specimens with different ages of old concrete and various surface roughness. The bonding surface for specimens in group B after splitting tensile tests is shown in Figure 3. It was discovered that when the age of the old concrete was 3 days, the failure surface of the specimen was accompanied with a large number of cement slurry fractures, and there were aggregate breaks and falls off on the edge. With the growth of the age, the failure surface remained more intact and smooth. For specimen at 14 and 28 days, some areas of cement slurry were broken and the edges were seriously damaged, and the specimen at 60 and 300 days were only prone to the phenomenon of aggregate breaking and falling off at the corners, while other areas are basically intact. 

**Figure 4:**
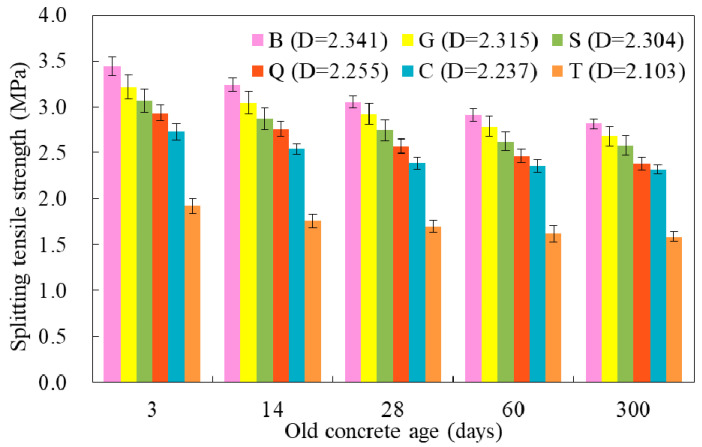
The splitting tensile failure with different ages of old concrete.

The splitting tensile strength results are shown in Figure 4. For the specimens in group B, G, S, Q, and C, the splitting tensile strength decreased as the increase of old concrete age. For the specimens in group T, the splitting tensile strength did not decrease significantly with the age, and the strength was the lowest in each phase. It was mainly due to lower roughness with small value of fractal dimension (2.103), which was more close to the smooth surface. At the same age of old concrete, the splitting tensile strength increased with the increasing surface roughness (higher fractal dimension). When the age of old concrete reached a certain age (60 days), the splitting tensile strength did not change significantly. When the age of the old concrete was shorter, the failure surface was accompanied with more cement slurry fractures, and the corresponding splitting tensile strength was larger (Figure 3 and Figure 4).

**Figure 5:**
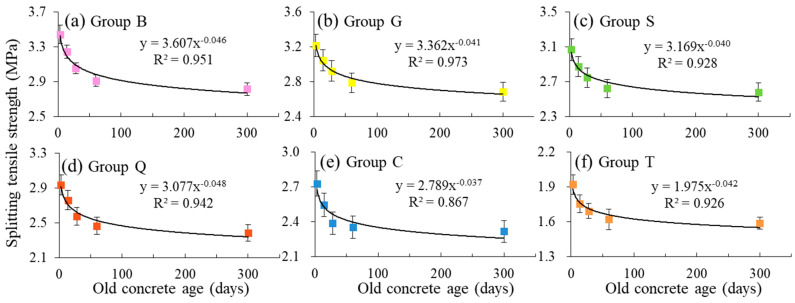
The relationship between splitting tensile strength and old concrete age for new-to-old concrete structure: (**a**) Group B, (**b**) Group G, (**c**) Group S, (**d**) Group Q, (**e**) Group C, (**f**) Group T.

In addition, there was a power function relationship between the splitting tensile strength and the age of the old concrete, as shown in Figure 5. The correlation coefficient (R^2^) was above 0.90 for six groups. It was confirmed that the old concrete age was an important factor to evaluate the internal defects of new-to-old concrete, which was related to new-to-old concrete strength closely. Furthermore, when the age of old concrete was less than 28 days, its influence on the interface strength of new and old concrete was more obvious.

According to the interfacial bonding mechanism of new-to-old concrete, interfacial bonding force mainly includes mechanical force, van der Waals force and chemical force. The mechanical forces include the mechanical bite force caused by the radiation growth of cement hydration products on the interface to the old concrete surface and the physical mechanical meshing force caused by the surface roughness. Van der Waals forces mainly refer to the forces caused by the interaction of crystal molecules in cement matrix. The chemical force is mainly formed by the chemical reaction between the cement hydration products in the old concrete and the interface agent. For the specimens in group T in this work, due to the small interface roughness, the interface adhesion force mainly came from the mechanical bite force and chemical force formed by the radiation growth of cement hydration products in the new concrete and the old concrete onto each other’s concrete surface. After 28 days, the cement hydration of the concrete was nearly completed, and the rate of the cement hydration reaction slowed down (Figure 5). When the age of the old concrete reached 28 days (28 days or more), the new concrete specimen were poured, and the binding force mainly came from the mechanical bite force generated by the radiation of cement hydration products in the new concrete to the old concrete surface. The cohesive force formed was small, and the age of the old concrete had little influence on the splitting tensile strength. At this stage, the cement slurry connection between the new concrete and the old concrete was less, and the failure surface was more complete. Therefore, if the new concrete was poured before the completion of the old concrete hydration, the interface bonding formed was larger (Figure 4 and Figure 5).

### 3.3. Influence of the Surface Roughness

**Figure 6:**
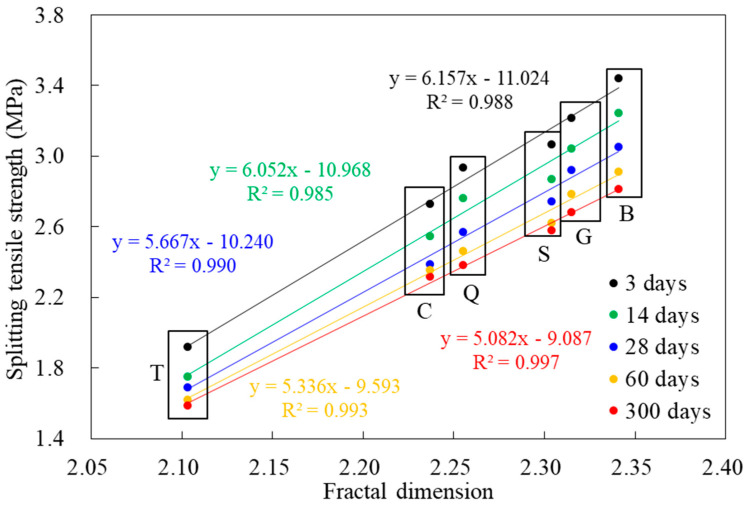
The variation law of splitting tensile strength with fractal dimension value D.

The splitting tensile strength of new-to-old concrete specimen with different interface roughness was shown in Figure 6. It was discovered that as the interface fractal dimension *D* increased, the splitting tensile strength increased correspondingly, and the growth law was linear in general. The correlation coefficient (R^2^) was above 0.98. The mechanical force played a key role in the interface adhesion of the new-to-old concrete, and the larger the fractal dimension *D*, the larger the bonding area and the greater the mechanical force. It also confirmed that the fractal analysis was a feasible method to evaluate the splitting tensile strength of new-to-old concrete. Furthermore, the results had an important guiding significance for the improvement of new-to-old concrete structure.

**Figure 7:**
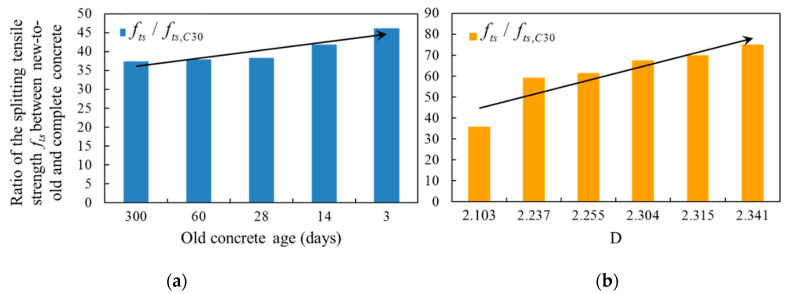
Comparison of influence of roughness and old concrete age on the splitting tensile strength. (**a**) The effect of age; (**b**) The effect of roughness.

Since the roughness and the age of the old concrete both had influence on the interfacial bonding, the splitting tensile strength of the new-to-old concrete under the influence of these two factors were compared with the splitting tensile strength of the complete specimen of C30 in Figure 7. The average splitting tensile strength of the specimen with different interface roughness was 31.01% to 64.71% of the splitting tensile strength of C30, and the variation range was 33.7%; while for the specimens with different old concrete age was 55.27% to 69.68% with the variation of 14.41% (group G), and 31.01% to 39.88% with the variation of 8.87% (group T). When the roughness of the interface was changed, the variation range of the tensile strength was more than 30%, while when the age of old concrete was changed, the change range of splitting tensile strength was no more than 15%. Therefore, the interface roughness had an obvious influence on the interface bond performance, and the age of old concrete also had an influence on the interface bond, but the influence was not obvious, especially the age of old concrete exceeds 28 days. 

### 3.4. Ultrasonic Non-Destructive Test Results

**Figure 8:**
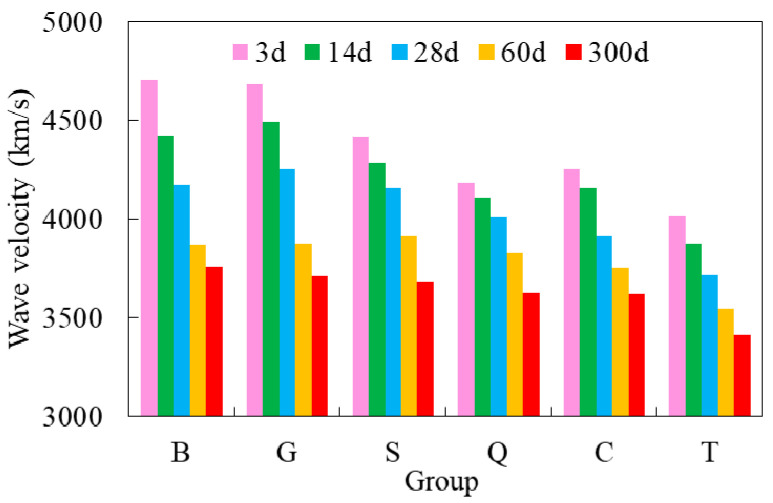
Results from ultrasonic non-destructive test.

Figure 8 shows the ultrasonic non-destructive test results. The wave velocities of group G and B were the highest at each curing age. Under the same surface roughness condition, the wave velocity decreased obviously with the increase of the age of the old concrete. Under the same age of the old concrete (less 28 days), the wave velocity was closely related to the age of the roughness. When the old concrete age was longer than 28 days, the surface roughness had little effect on wave velocity. The results corresponded to the mechanical properties well, which could be understood that the ultrasonic non-destructive test results could reflect the interior defects of concrete such as pores and micro cracks. The effect of surface roughness and age of old concrete on the bond surface was well proved by ultrasonic non-destructive test.

**Figure 9:**
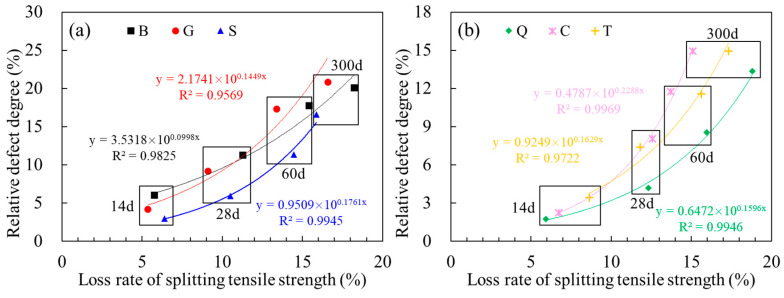
The internal defects of new-to-old concrete: (**a**) Group B, G, and S; (**b**) Group Q, C, and T.

The mechanical properties of concrete is mainly affected by internal defects, and the relative defect degree *D*_R_ is characteristic parameter, which can be used to evaluate the strength [36,37]. The relationship considered the influence of surface roughness and old concrete age is further analyzed. The results are shown in Figure 9, where loss rate of splitting tensile strength is defined as Equation (5) [36]:

(5) $$L=\frac{f_{ts-3}-f_{ts-t}}{f_{ts-3}}\times 100\%$$

where *L* is the loss rate of splitting tensile strength (%), *f_ts-t_* is the splitting tensile strength with old concrete at *t* days (km/s), which satisfied *t* > 3, *f_ts-3_* is the splitting tensile strength with 3 days of old concrete (MPa).

In Figure 9, the relative defect degree (*D*_R_) increased with old concrete curing days, which represented the degradation of the internal density of the concrete correspondingly. The loss rate of splitting tensile strength was also increased with longer age of old concrete. It could be seen that the relative defect degree (*D*_R_) met the exponential relationship with the loss rate of splitting tensile strength, and the correlation coefficient *R*^2^ arranged from 0.9569 to 0.9969 (Figure 9). The results showed that the relative defect degree characterization method proposed in this paper could be used to evaluate the mechanical properties of concrete. In combination with the results of ultrasonic non-destructive test, the loss rate of splitting tensile strength could be evaluated, and the long-term strength of new-to-old concrete could be also predicted further based on the early strength.

### 3.5. Resistance to F-T Cycles

**Figure 10:**
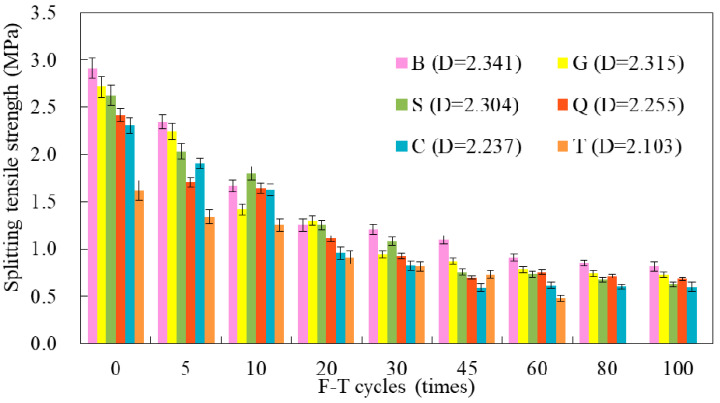
The splitting tensile strength of new-to-old concrete at 28 days under F-T cycles.

The splitting tensile strength results of the new-to-old concrete with F-T cycles are shown in Figure 10. It could be seen that the splitting tensile strength of the new-to-old concrete with higher surface roughness was larger than that of specimens with lower surface roughness during the whole F-T cycles. After 60 times of F-T cycles, the specimens in group B lost most of the bonding force, and the splitting tensile strength maintained at a low level, which accounted for 25–30% of the initial strength approximately. For group T, there was no bonding force after 60 times of F-T cycles, and the other specimens with interfacial roughness still had a certain degree of bonding force.

**Figure 11:**
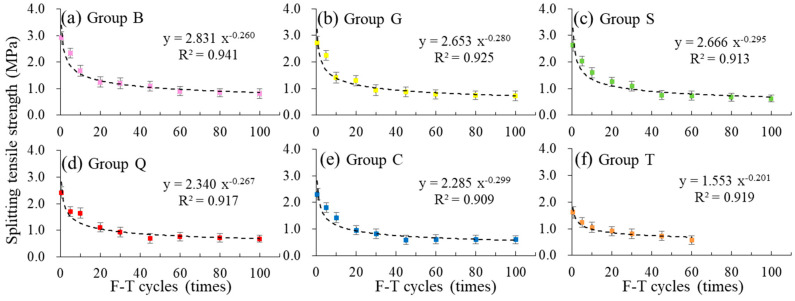
The relationship between splitting tensile strength and the times of F-T cycles: (**a**) Group B, (**b**) Group G, (**c**) Group S, (**d**) Group Q, (**e**) Group C, (**f**) Group T.

The relationships between splitting tensile strength and the times of F-T cycles are shown in Figure 11. With the increase of F-T cycles, the splitting tensile strength increased as power function, and the specimens with different interface roughness met the relationship generally. The correlation coefficient R^2^ arranged from 0.909 to 0.941. The splitting tensile strength of specimens showed an obvious two-stage change rule under F-T cycles. In the early stage of F-T cycles the strength dropped rapidly, and in the later period the loss of strength tended to be slow.

**Figure 12:**
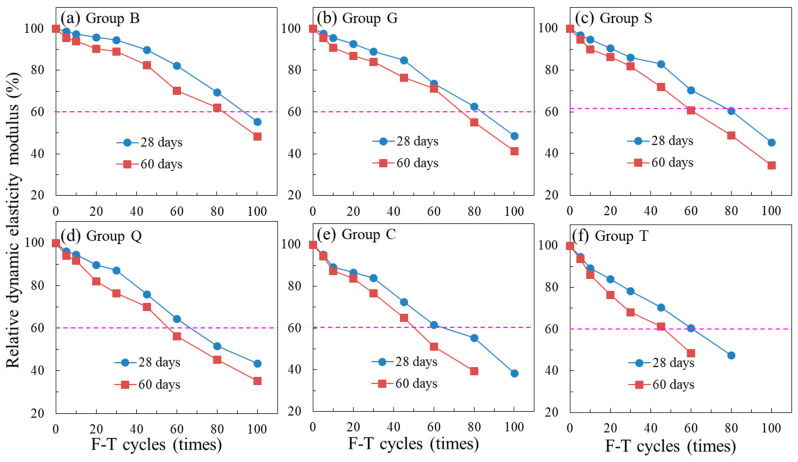
The relative dynamic elasticity modulus of new-to-old concrete under F-T cycles: (**a**) Group B, (**b**) Group G, (**c**) Group S, (**d**) Group Q, (**e**) Group C, (**f**) Group T.

The relative dynamic elasticity modulus of new-to-old concrete under F-T cycles are shown in Figure 12. The relative dynamic elasticity modulus decreased obviously under F-T cycles. When the relative dynamic elasticity modulus decreased by 40%, the F-T resistance of specimens failed. It could be seen that the specimens with large roughness (Group B and G) and shorter old concrete curing ages (28 days and 60 days) had a better F-T resistance, and the influence of interface roughness was more obvious than that of old concrete ages.

**Figure 13:**
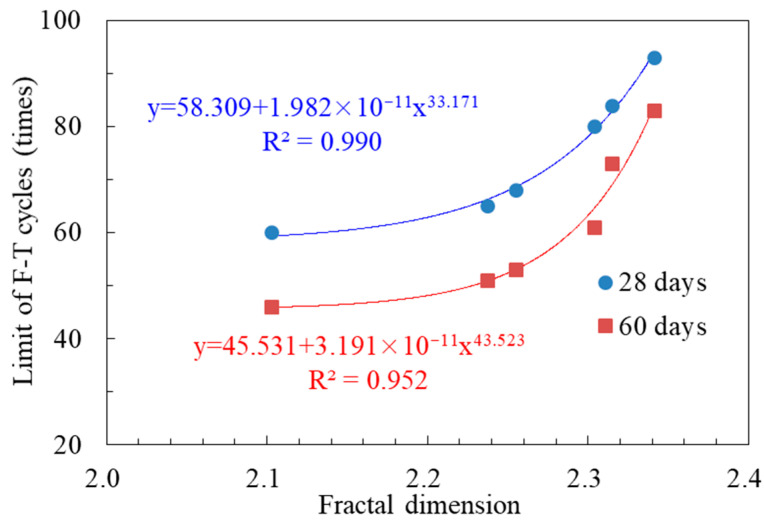
The relationships between surface fractal dimension and limit of F-T cycles.

The relationship between surface fractal dimension and limit of F-T cycles is shown in Figure 13, where the limit of F-T cycles refers to the maximum times of F-T cycles that the specimens can withstand (F-T resistance). It could be seen that the development of F-T cycles limit met the power function relationship with the increase of fractal dimension, and the correlation coefficient R^2^ was greater than 0.95. This relationship was also satisfied with specimens of different old concrete curing ages. Moreover, when the fractal dimension increased from 2.1 to 2.2, the F-T resistance of the new-to-old concrete did not increase obviously, and when the fractal dimension was above 2.3, the increase of interface roughness could improve the F-T resistance significantly.

For the new and old concrete specimens with large interface roughness, the mechanical force not only comes from the strong mechanical biting force formed by the radiation growth of cement hydration products, but also plays a major role in the physical mechanical meshing force formed by the surface roughness. For the F-T attack, the mechanical biting force of the specimens with rough interface was destroyed by freeze-thaw reaction products. The interface will not separate because the physical mechanical meshing force formed by roughness still exists between the interfaces under the action of freeze-thaw, so that the interface still maintains a low level of bonding force under the action of freeze-thaw. For the specimens with smoother interface, the bonding force is mainly generated by the radiation growth of cement hydration reaction products, which is destroyed by freeze-thaw action. The physical and mechanical meshing force formed by the rough surface is basically lower, and the bonding force is also lower. The concrete on both sides of the interface is easier to separate from each other.

## 4. Conclusions

Based on the experimental investigation and the discussion of results, the following conclusions are drawn:

(1) The larger roughness and the smaller age of old concrete can improve the bond performance, and the effect of roughness on the bonding property of the new-to-old concrete structure is more significant than that of old concrete age. The influence of roughness, old concrete age and F-T cycles on the splitting tensile strength of new-to-old concrete was analyzed quantitatively.
(2) Fractal dimension *D* can characterize the adhesive interface roughness well, and the splitting tensile strength changes linearly with the fractal dimension *D*. The relationship between F-T resistance of new-to-old concrete and fractal dimension *D* satisfy a power function.
(3) The ultrasonic non-destructive test can describe the defect situation of the bonding interface, and for the new-to-old concrete specimens with different old concrete ages and interface roughness, the relative defect degree has an exponential relationship with the loss rate of splitting tensile strength.
(4) Small interface roughness cannot improve the F-T resistance of the new-to-old concrete obviously, and when the fractal dimension of interface is above 2.3, the improvement becomes significant.

## Figures and Tables

**Table 1:** Concrete composition of the old concrete and new concrete.

Materials	Water Cement Ratio	Cement kg/m^3^	Sand kg/m^3^	Gravel kg/m^3^	Waterk g/m^3^
Old concrete (C30)	0.52	375	641	1189	195
New concrete (C35)	0.47	415	609	1181	195

## Data Availability

Data is contained within the article.
